# The establishment of PD-1 inhibitor treatment prognosis model based on dynamic changes of peripheral blood indexes in patients with advanced lung squamous cell carcinoma

**DOI:** 10.3389/fonc.2024.1454709

**Published:** 2024-12-17

**Authors:** Yuyan Xie, Hao Sun, Liying Shan, Xin Ma, Qingyu Sun, Fang Liu

**Affiliations:** ^1^ Department of Medical Oncology, Harbin Medical University Cancer Hospital, Harbin, China; ^2^ Department of Gastrointestinal Surgery, Harbin Medical University Cancer Hospital, Harbin, China

**Keywords:** peripheral blood indexes, advanced LUSC, immunotherapy, PD-1 inhibitor, squamous cell carcinoma antigen, neuron-specific enolase

## Abstract

**Background:**

Unlike patients with lung adenocarcinoma, patients with lung squamous cell carcinoma (LUSC) do not derive significant benefits from targeted therapy. In recent years, immunotherapy has revolutionized the treatment approach for LUSC. However, not all patients with this type of cancer respond to immunotherapy, necessitating the identification of effective biomarkers to predict survival prognosis and evaluate the efficacy of PD-1 inhibitors.

**Materials and methods:**

We retrospectively collected case and hematologic data from 212 patients with advanced squamous lung cancer who received PD-1 combination therapy. Hematological indices mainly contained SCC, CEA, NSE, Hb, LDH, WBC and RBC at baseline, 6 and 12 weeks of treatment. All patients underwent imaging examinations and efficacy was evaluated according to RECIST1.1 criteria. Univariate tests were used to assess the relationship between changes in serum biomarkers, clinical characteristics and treatment outcome. The survival prognosis of patients was investigated by telephone follow-up. The optimal critical values of all hematological indicators were calculated by ROC curves, and then logistic regression and Cox regression were used to analyze multiple serum markers in relation to efficacy and survival prognosis, respectively. Finally, column line plots were constructed and validated to predict the probability of patient survival.

**Results:**

Post-treatment RBC_12w_<3.81 × 10 ^12^/L (*p* < 0.034) was associated with lower ORR, and WBC_6w_<9.34 × 10^9^/L (*p*=0.041) was associated with higher DCR.SCC_12w_≥2.25 ng/mL (*p* = 0.015), NSE_6w_≥13.54 ng/mL(*p* = 0.044)and RBC_0w_≥4.2 × 10 ^9^/L (*p* = 0.003) were independent predictors of PFS. SCC_12w_≥2.25 ng/mL (*p* < 0.001) and NSE_6w_≥13.54ng/mL(*p* = 0.042) were independent predictor of OS. Patients in the SCC_12w_≥2.25 ng/mL (HR = 1.943,95% CI:1.218-3.079 vs. HR = 2.161,95%CI:1.087-3.241) and NSE_6w_≥13.54 ng/mL (HR = 1.657,95% CI:1.118-2.535 *vs*. HR = 2.064,95% CI:1.569-4.169) groups had shorter PFS and OS. In subgroup analysis, patients with stage III advanced squamous lung cancer had a better pro-gnosis than those with stage IV. PD-L1-positive, and SCC_12w_ ≥2.25 ng/mL had a worse prognosis. The results of constructing column-line plots for predicting the survival probability of 1-, 3-, and 5-year PFS and OS: The C-index and 95% CI for PFS and OS of column-line plots were 0.725 (95% CI: 0.478-1.928) and 0.755 (95% CI: 0.642-0.868), respectively, and the bootstrap correction showed a good consistency of the column-line plots.

**Conclusion:**

Changes in RBC_12w_ ≥3.81×10^12^/L, WBC_6w_ ≥9.34×10 ^9^/L, SCC_12w_ ≥2.25 ng/mL, and NSE_6w_ ≥13.54 ng/mL after treatment are prognostic indicators of immunotherapy in patients with advanced squamous lung cancer.

## 1 Introduction

Lung cancer has one of the highest incidences and mortality rates globally, with non-small cell lung cancer (NSCLC) comprising approximately 80-85% of all lung cancer cases (1). Limited progress has been achieved in the treatment of squamous lung cancer over the past few decades, and platinum-based chemotherapy continues to be the primary first-line treatment option (2). Due to significant advancements in immunotherapy, PD-1 inhibitors have gained widespread usage in patients diagnosed with squamous lung cancer. Nevertheless, the efficacy of immunotherapy for advanced NSCLC in unselected populations is merely 20%-40% (3), and a subset of patients may experience severe adverse events or expedited mortality due to tumor hyper-progression. The U.S. Food and Drug Administration has authorized the use of PD-L1 expression and tumor mutational load as clinical prognostic indicators for survival in patients undergoing treatment with immune checkpoint inhibitors (ICIs) (4, 5). However, the clinical application of these predictive biomarkers is restricted due to their high cost, complexity, and time delay in obtaining results. Furthermore, cancer immunity undergoes continuous changes during immunotherapy, and the repeated utilization of tissue biopsies for longitudinal immunoassays is not clinically viable, particularly in cases of rapid clinical deterioration. Blood-based immune biomarkers can overcome these limitations associated with tissue-based immunomarkers in tumor immunotherapy, since peripheral blood sampling is readily accessible, minimally invasive, and reproducible.

In recent years, blood cell counts (6), peripheral blood inflammation indicators (7, 8), and tumor markers (such as CEA, NSE, CA129, and SCC) (9, 10) have gained widespread utilization in predicting the prognosis of chemotherapy or targeted therapy in NSCLC. Apart from PD-L1, the investigation of inflammatory biomarkers, including the neutrophil/lymphocyte ratio (NLR) and lactate dehydrogenase (LDH), is still at an early stage (11). The objective of this study was to retrospectively evaluate the correlation between dynamic changes in peripheral blood and prognosis among patients diagnosed with squamous lung cancer who underwent immunotherapy. Moreover, pertinent clinical prediction models were developed to provide guidance for the implementation of immunotherapy in clinical settings.

## 2 Materials and methods

### 2.1 Research design

We conducted a retrospective study of patients diagnosed with inoperable locally advanced or advanced squamous cell lung cancer who were treated with PD-1 inhibitors between January 2018 and July 2023 at the Harbin Medical University Cancer Hospital. All patients and clinical information were analyzed according to the Declaration of Helsinki and its amendments. The following inclusion criteria were applied:(1) Patients were administered intravenously every three weeks, with a routine therapeutic dose of 200 mg (240 mg for Teraplizumab) of PD-1 inhibitors. (2) In combination with/without combination of chemotherapeutic or antiangiogenic agents, with chemotherapeutic agents mainly being albumin paclitaxel, gemcitabine, docetaxel, cis/carboplatin, and antiangiogenic drugs mainly bevacizumab, erlotinib, and endo;(3) Efficacy assessment every two cycles. The following patients were excluded: (I) unclear pathology or inability to obtain pathological tissues; (II) patients with LUSC amenable to surgical treatment; (III) imperfect imaging data (CT, MRI, ultrasound and PET/CT), missing data; (IV) accompanied by severe comorbidities (acute cerebral infarction, acute hepatic and renal failure, severe coronary artery disease, cardiac arrhythmia and so on); and (V) concomitant with other malignant tumors. Because this was a retrospective study, informed consent was waived by the Harbin Medical University Cancer Hospital Ethics Committee.

Baseline covariates encompassed patients’ age, gender, smoking history, Eastern Cooperative Oncology Group performance status assessment (ECOG score), clinical stage, history of radiotherapy, history of surgery, PD-L1 expression, treatment modality (monotherapy, chemotherapy combined with immunotherapy, anti-angiogenesis + immunotherapy, chemotherapy + immunotherapy + anti-angiogenesis), number of lines of immunotherapy (1, ≥2), presence of distant metastases (liver, bone, brain), adverse reactions to immunotherapy, time to disease progression, and short-term efficacy assessment. Furthermore, peripheral blood markers such as SCC, CEA, NSE, Hb, LDH, WBC, and RBC were regularly assessed at weeks 0, 6, and 12 during the course of immunotherapy.

### 2.2 Research endpoints

The primary endpoints were the objective response rate (ORR) and the disease control rate (DCR), while survival outcomes were assessed using progression-free survival (PFS) and overall survival (OS). ORR was defined as the proportion of patients achieving CR (CR) or partial remission (PR), while DCR was defined as the proportion of patients achieving CR, PR, or stable disease (SD). Additionally, ORR and DCR were evaluated based on RECIST 1.1 criteria. For patients without evidence of disease progression, PFS was censored at the time of the last follow-up. OS was defined as the time from diagnosis to either death or the last follow-up.

### 2.3 Statistical analysis

Statistical analysis was performed using SPSS 27.0 and R4.3.2 software, and graphs were plotted using GraphPad Prism 10.0. A two-sided *P* < 0.05 was considered statistically different. The Kolmogorov-Smirnov test was used to determine whether continuous variables conformed to a normal distribution. For continuous variables that conformed to normal distribution, the mean ± standard deviation (*x ± s*) was used. For continuous variables that did not conform to normal distribution, median and interquartile range (IQR) were used. Categorical variables were expressed using the number of cases and percentage (n, %). In order to study the influence of peripheral blood on prognosis, we divided the indexes of peripheral blood into two parts according to the critical value of ROC curve. For survival outcomes, we calculated the relationship between peripheral blood indices of optimal treatment response by multifactorial logistic regression. We used the Kaplan-Meier method to generate PFS and OS survival curves and Log-rank tests for survival outcomes in patients with each factor. Relative risk was assessed by risk ratios and 95% confidence intervals. In addition, we constructed Cox risk-proportional models to analyze independent prognostic factors. Factors with *P* < 0.05 in the univariate Cox regression analysis were further included in the multivariate analysis to identify factors independently associated with survival. Finally, we tested the proportional risk hypothesis by the Schenfeld residual method for indicators that were significant in the multivariate analyses and created column-line plots to predict the probability of patient survival. In addition, the accuracy of the column-line plots was predicted by plotting calibration curves.

## 3 Results

### 3.1 Patient characteristics

This study included a total of 212 patients diagnosed with advanced squamous lung cancer. **Supplementary Table 1** presents the baseline characteristics of the patients. The patients had a median age of 61.33 years, with approximately 85.8% being male. Among the patients, 63.7% were smokers, and the majority (94.4%) had ECOG scores ranging from 0 to 1, while only 5.6% had scores ≥2. Among the 212 patients with squamous lung cancer, all patients received PD-1 inhibitor-based therapy. Among them, 170 patients received first-line immunization therapy, and 19.8% received immunization therapy after multiple lines of treatment. The most commonly used PD-1 inhibitor, Tislelizumab, accounted for approximately 56.6% of the patients, while the remaining patients were treated with Pembrolizumab (18.9%) and Sintilimab (17%). Additionally, PD-L1 expression was evaluated in only 81 patients. Among them, 4.2% tested negative for PD-L1, 34% had received radiotherapy.

### 3.2 Hematologic parameters

The median levels of SCC, CEA, Hb, LDH, NSE, RBC, and WBC at 0, 6, and 12 weeks were as follows: 1.50 ng/mL, 1.00 ng/mL, 1.10 ng/mL, 3.17 ng/mL, 3.18 ng/mL, 3.14 ng/mL, 137.00 g/L, 128.50 g/L, 125.00 g/L, 188.50 U/L, 188.00 U/L, 189.50 U/L, 14.88 ng/mL, 13.36 ng/mL, 12.70 ng/mL, 7.36×10^9^/L, 6.48×10^9^/L, 6.16×10^9^/L, 4.58×10^12^/L, 4.28×10^12^/L, 4.01×10^12^/L. Detailed information is shown in **Supplementary Table 1**.

### 3.3 Optimal cut-off values

The optimal cutoffs for SCC, CEA, NSE, Hb, LDH, WBC, and RBC at 0, 6, and 12 weeks were obtained by using the maximum dominance index [sensitivity - (1 - specificity)] calculated from the subjects’ work characteristic curves. In addition, area under the curve analysis was performed and found that SCC_12w_ had the highest area under the curve among the peripheral blood indices. The details are shown in the **Supplementary Table 2** and **Supplementary Figure 1**.

### 3.4 Multivariate logistic regression analysis of DCR and ORR

To predict the correlation between peripheral blood markers and response to PD-1 inhibitor therapy, we used multivariate logistic regression, calculated as


$$\text{logit}\left(p\right)=\ln\left(\frac{p}{1-p}\right)=\beta_{0}+\beta_{1}x_{1}+\beta_{2}x_{2}+\cdots +\beta_{n}x_{n}\;$$

We found that RBC_12w_<3.81×10 ^12^/L (HR = 0.405, 95% CI: 0.155-0.971, *p* = 0.034) was associated with a lower ORR and WBC_6w_<9.34× 10 ^9^/L (HR = 2.510, 95% CI: 1.997-6.322, *p* = 0.041) was associated with higher DCR. (**Supplementary Table 3**)

### 3.5 Univariate and multifactorial Cox analysis

Univariate analysis identified ECOG score (0-1), Treatment, Radiotherapy, SCC levels (SCC_0w_, SCC_6w_, SCC_12w_), NSE levels (NSE_0w_, NSE_6w_, NSE_12w_), LDH_12w_, and RBC_0w_ at baseline and post-treatment as significant prognostic factors for both PFS and OS. Among these, ECOG score, Treatment, Radiotherapy, SCC_12w_, NSE_6w_, and RBC_0w_ were specifically associated with PFS, while Treatment, CEA levels (CEA_0w_, CEA_6w_), NSE_6w_, Hb levels (Hb_0w_, Hb_12w_), and LDH levels (LDH_6w_, LDH_12w_) were associated with OS.

**Figure 1:**
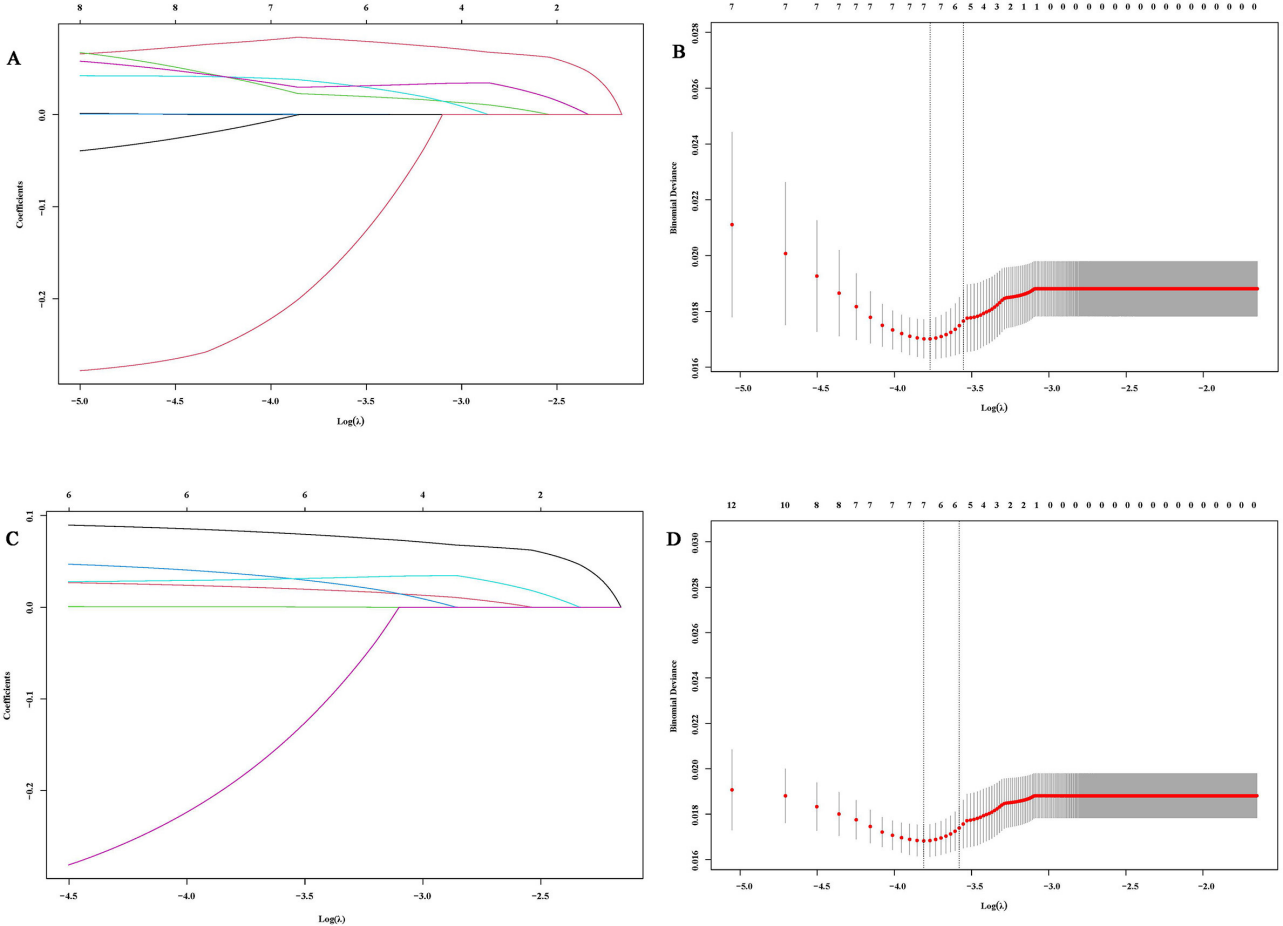
Variable selection based on Lasso regression. **(A, C)** Characteristic changes in the coefficients of variables for PFS and OS; **(B, D)**. The process of selecting the optimal value of parameter λ in the Lasso regression model through cross-validation methods.

To address potential multicollinearity, significant factors from the univariate analysis were further screened using Lasso regression. For PFS analysis, when the optimal λ was taken to be a value of 0.0047, SCC_6w_ and NSE_12w_ were excluded due to multicollinearity (**Figures 1A, B**). Similarly, when taking the optimal λ value of 0.0056, in OS analysis, SCC_0w_, SCC_6w_, CEA_6w_, Hb_0w_, Hb_12w_, LDH_6w_, and RBC_0w_ were excluded (**Figures 1C, D**).

Multivariate analysis ultimately revealed that Treatment, SCC_12w_, and NSE_6w_ were independent prognostic factors for both PFS and OS. Additionally, RBC_0w_ was identified as an independent predictor for PFS. Detailed information is shown in **Tables 1**, **2**.

**Table 1:** Univariate and multivariate analyses for PFS.

Parameters	PFS			
	Univariate analysis	P value	Multivariate analysis	P value
	Hazard ratio (95%CI)		Hazard ratio (95%CI)	
Sex (Male *vs* Female)	1.089 (0.412-2.820)	0.860		
Age (<61 *vs*.≥61)	0.990 (0.944-1.038)	0.675		
Smoking (No *vs* Yes)	1.047 (0.526-2.087)	0.895		
ECOG					
0-1	0.094 (0.010-0.848)	0.035		
2	0.160 (0.021-1.197)	0.074		
4	0.492 (0.055-4.426)	0.527		
TNM Stage (III *vs* IV)	0.654 (0.305-1.404)	0.276		
Treatment (First line *vs* Non-first line)	0.129 (0.065-0.257)	<0.001	0.131 (0.060-0.286)	<0.001
Radiation (No *vs* Yes)	0.437 (0.222-0.860)	0.017		
Surgery (No *vs* Yes)	0.705 (0.306-1.622)	0.411		
SCC_0w_					
<3.15 ng/mL	1		1	
≥3.15 ng/mL	3.028 (1.012-1.044)	0.001	1.015 (0.994-1.037)	0.161
SCC_6w_					
<1.25 ng/mL	1			
≥1.25ng/mL	2.058 (1.032-1.085)	<0.001		
SCC_12w_					
<2.25 ng/mL	1		1	
≥2.25 ng/mL	3.062 (2.036-4.088)	<0.001	1.943 (1.218-3.079)	0.015
CEA_0w_					
<4.55 ng/mL	1			
≥4.55 ng/mL	1.000 (0.994-1.006)	0.976		
CEA_6w_					
<3.41 ng/mL	1			
≥3.41 ng/mL	1.003 (0.998-1.008)	0.286		
CEA_12w_					
<5.34 ng/mL				
≥5.34 ng/mL	1.004 (0.995-1.012)	0.365		
NSE_0w_					
<22.30 ng/mL	1		1	
≥22.30 ng/mL	1.947 (1.012-1.083)	0.008	1.013 (0.958-1.072)	0.652
NSE_6w_					
<13.54 ng/mL	1		1	
≥13.54 ng/mL	2.087 (1.346-3.129)	<0.001	1.657 (1.118-2.535)	0.044
NSE_12w_					
<14.55 ng/mL	1			
≥14.55 ng/mL	3.075 (1.030-1.122)	0.001		
Hb_0w_					
<119.50 g/L	1			
≥119.50 g/L	0.984 (0.968-1.000)	0.056		
Hb_6w_					
<117.50 g/L	1			
≥117.50 g/L	0.987 (0.966-1.008)	0.220		
Hb_12w_					
<173.00 g/L	1			
≥173.00 g/L	0.988 (0.971-1.005)	0.151		
LDH_0w_					
<187.50 U/L	1			
≥187.50 U/L	1.002 (0.997-1.007)	0.386		
LDH_6w_					
<241.50 U/L	1			
≥241.50 U/L	1.602 (0.999-1.005)	0.135		
LDH_12w_					
<191.50 U/L	1			
≥191.50 U/L	1.701 (1.000-1.003)	0.023		
WBC_0w_					
<5.39×109/L	1			
≥5.39×109/L	0.942 (0.811-1.093)	0.430		
WBC_6w_					
<9.34×109/L	1			
≥9.34×109/L	1.201 (0.876-1.144)	0.985		
WBC_12w_					
<5.88×109/L	1			
≥5.88×109/L	1.059 (0.922-1.216)	0.418		
RBC_0w_					
<5.81×10^12^/L					
≥5.81×10^12^/L	0.495 (0.274-0.896)	0.020	0.518 (0.283-0.947)	0.033
RBC_6w_					
<4.20×10^12^/L	1			
≥4.20×10^12^/L	0.883 (0.500-1.559)	0.667		
RBC_12w_					
<3.81×10^12^/L	1			
≥3.81×10^12^/L	1.063 (0.662-1.706)	0.800		

**Table 2:** Univariate and multivariate analyses for OS.

Parameters	OS			
	Univariate analysis	P value	Multivariate analysis	P value
	Hazard ratio (95%CI)		Hazard ratio (95%CI)	
Sex (Male *vs* Female)	1.225 (0.471-3.189)	0.677		
Age (<61 *vs*.≥61)	0.990 (0.941-1.041)	0.682		
Smoking (No *vs*. Yes)	1.359 (0.671-2.753)	0.394		
ECOG				
0-1	0.735 (0.076-7.140)	0.791		
2	1.257 (0.151-10.446)	0.832		
4	2.780 (0.273-28.292)	0.388		
TNM Stage (III *vs* IV)	1.695 (0.805-3.567)	0.165		
Treatment (First line *vs* Non-first line)	2.289 (1.163-4.503)	0.016	2.143 (1.002-4.582)	0.049
Radiation (No *vs* Yes)	0.760 (0.385-1.499)	0.760		
Surgery (No *vs* Yes)	0.502 (0.217-1.165)	0.502		
SCC_0w_				
<3.15 ng/mL	1			
≥3.15 ng/mL	1.018 (1.003-1.032)	0.016		
SCC_6w_				
<1.25 ng/mL	1			
≥1.25ng/mL	2.130 (1.076-1.187)	<0.001		
SCC_12w_				
<2.25 ng/mL	1			
≥2.25 ng/mL	3.146 (2.076-5.210)	<0.001	2.161 (1.087-3.241)	<0.001
CEA_0w_				
<4.55 ng/mL	1			
≥4.55 ng/mL	1.724 (1.403-3.046)	0.026	0.977 (0.942-1.015)	0.234
CEA_6w_				
<3.41 ng/mL	1			
≥3.41 ng/mL	1.532 (1.365-2.013)	0.049		
CEA_12w_				
<5.34 ng/mL	1			
≥5.34 ng/mL	1.005 (0.995-1.015)	0.333		
NSE_0w_				
<22.30 ng/mL	1		1	
≥22.30 ng/mL	1.086 (1.036-1.139)	0.001	1.073 (0.996-1.156)	0.065
NSE_6w_				
<13.54 ng/mL	1		1	
≥13.54 ng/mL	3.087 (1.943-5.133)	<0.001	2.064 (1.569-4.169)	0.042
NSE_12w_				
<14.55 ng/mL	1			
≥14.55 ng/mL	1.032 (0.992-1.073)	0.117		
Hb_0w_				
<119.50 g/L	1			
≥119.50 g/L	0.781 (0.563-0.999)	0.036		
Hb_6w_				
<117.50 g/L	1			
≥117.50 g/L	0.980 (0.958-1.002)	0.076		
Hb_12w_				
<173.00 g/L	1			
≥173.00 g/L	0.581 (0.365-0.798)	0.029		
LDH_0w_				
<187.50 U/L	1			
≥187.50 U/L	1.003 (0.999-1.007)	0.103		
LDH_6w_				
<241.50 U/L	1			
≥241.50 U/L	2.323 (1.875-3.006)	0.023		
LDH_12w_				
<191.50 U/L	1		1	
≥191.50 U/L	1.881 (1.600-3.503)	0.036	1.001 (0.999-1.002)	0.260
WBC_0w_				
<5.39×10^9^/L	1		1	
≥5.39×10^9^/L	1.041 (0.893-1.214)	0.604	0.865 (0.730-1.024)	0.192
WBC_6w_				
<9.34×10^9^/L	1			
≥9.34×10^9^/L	1.008 (0.905-1.123)	0.883		
WBC_12w_				
<5.88×10^9^/L	1			
≥5.88×10^9^/L	1.079 (0.939-1.239)	0.283		
RBC_0w_				
<5.81×10^12^/L	1			
≥5.81×10^12^/L	0.560 (0.316-0.991)	0.047		
RBC_6w_				
<4.20×10^12^/L	1			
≥4.20×10^12^/L	0.812 (0.444-1.484)	0.498		
RBC_12w_				
<3.81×10^12^/L	1			
≥3.81×10^12^/L	0.851 (0.537-1.349)	0.749		

### 3.6 Survival analysis of SCC_12w_ and NSE_6w_

**Figure 2:**
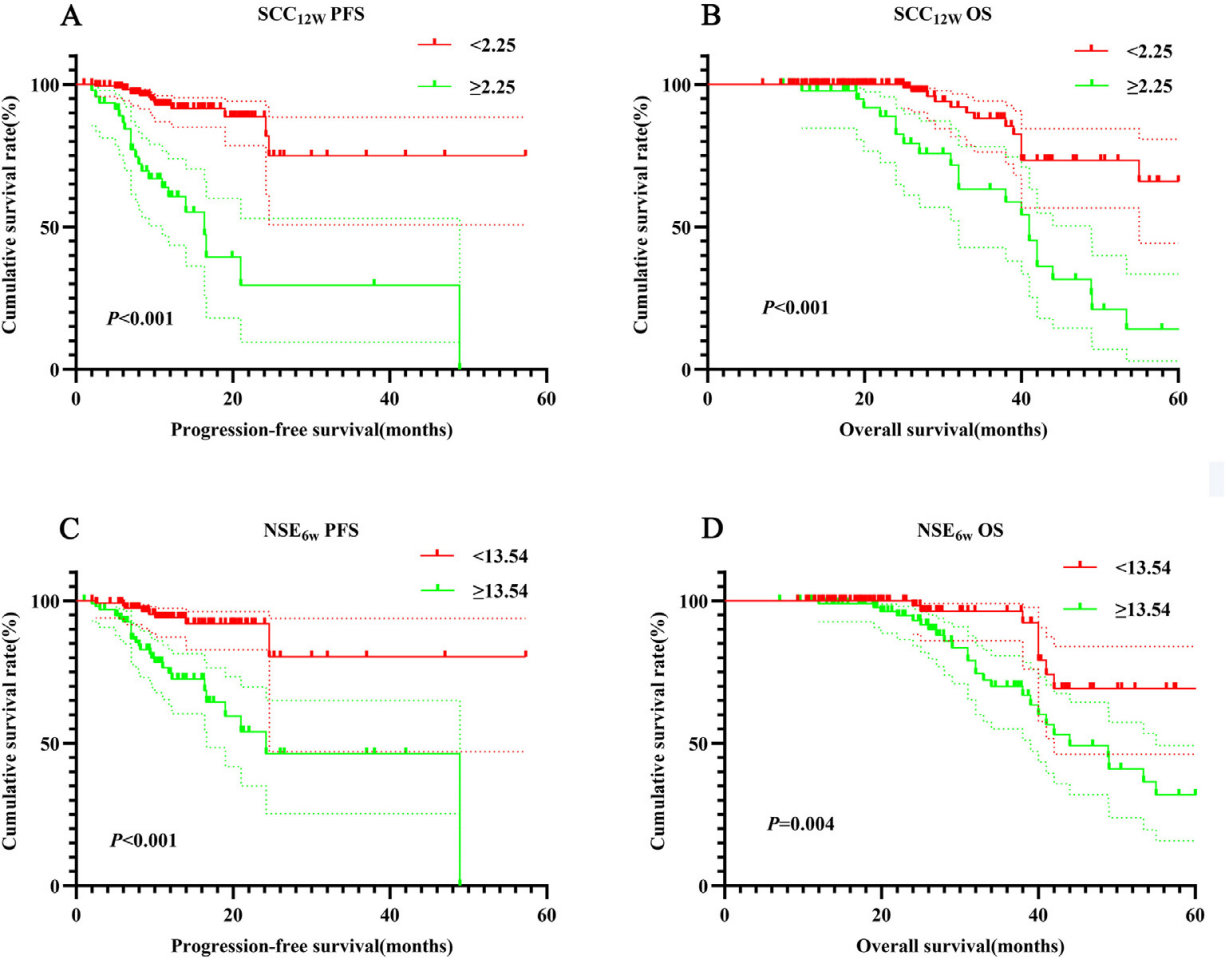
Survival curve for SCC_12w_ and NSE_6w_. SCC_12w_-ralated survival curve for **(A)** PFS and **(B)** OS; NSE_6w_-ralated survival curve for **(C)** PFS and **(D)** OS.

The 1- and 3-year PFS rates in the SCC_12w_ < 2.25 ng/mL group were 92.2% (95% CI:0.878-0.968) and 74.5% (95% CI:0.582-0.954), respectively, while the OS rates were 99.5% (95% CI:0.978-1.000) and 84.4% (95% CI:0.751-0.948). In contrast, the SCC_12w_ ≥ 2.25 ng/mL group exhibited significantly lower 1- and 3-year PFS rates of 62.0% (95% CI:0.482-0.796) and 30.2% (95% CI:0.136-0.670), with corresponding OS rates of 97.6% (95% CI:0.931-1.000) and 60.6% (95% CI:0.444-0.828). Similarly, in the NSE_6w_ < 13.54 ng/mL group, the 1- and 3-year PFS rates were 84.0% (95% CI:0.774-0.912) and 75.1% (95% CI:0.643-0.876), and the OS rates were 99.3% (95% CI:0.978-1.000) and 78.4% (95% CI:0.678-0.892). However, the NSE_6w_ ≥ 13.54 ng/mL group showed 1- and 3-year PFS rates of 88.3% (95% CI:0.809-0.964) and 58.0%(95% CI:0.382-0.880), and OS rates of 98.6%(95% CI:0.959-1.000) and 80.4%(95% CI:0.764-0.943). These findings indicate that patients with SCC_12w_ ≥ 2.25 ng/mL and NSE_6w_ ≥ 13.54 ng/mL experienced significantly shorter PFS and OS (**Figure 2**).

### 3.7 Treatment

In this study, 38 patients received PD-1 inhibitors alone (ICIs group), while 174 patients underwent chemotherapy combined with immunotherapy (ICIs+Chemo group). Baseline demographics and disease characteristics are summarized in **Table 3**. Significant differences were observed between the two groups regarding the number of treatment lines (*p* = 0.023), Hb_0w_ (*p* = 0.002), and WBC_12w_ (*p* = 0.047).

**Table 3:** The clinical characteristics for treatment (ICIs and ICIs+Chemo).

n	Level	ICIs (n=38)	ICIs+Chemo (n=174)	*p*
Age,Mean (SD)		59	62	
Sex, n (%)	Male	36 (94.7)	146 (83.9)	0.121
	Female	2 (5.3)	28 (16.1)	
Smoking, n (%)	Yes	22 (57.9)	113 (64.9)	0.458
	No	16 (42.1)	61 (35.1)	
ECOG, n (%)	0-1	36 (94.7)	163 (93.7)	1.000
	≥2	2 (5.3)	11 (6.3)	
TNM Stage, n (%)	III	14 (36.8)	45 ( (25.9)	0.230
	IV	24 (63.2)	129 (74.1)	
Treatment, n (%)	First line	25 (65.8)	145 (83.3)	0.023
	Non-first line	13 (34.2)	29 (16.7)	
Radiation, n (%)	Yes	14 (36.8)	59 (33.9)	0.711
	No	24 (63.2)	115 (66.1)	
SCC_0w_	<3.15 ng/mL	29 (76.3)	118 (67.8)	0.338
	≥3.15 ng/mL	9 (23.7)	56 (32.2)	
SCC_6w_	<1.25 ng/mL	20 (52.6)	111 (63.8)	0.204
	≥1.25ng/mL	18 (47.4)	63 (36.2)	
SCC_12w_	<2.25 ng/mL	26 (68.4)	135 (77.6)	0.294
	≥2.25 ng/mL	12 (31.6)	39 (22.4)	
CEA_0w_	<4.55 ng/mL	28 (73.7)	114 (65.5)	0.446
	≥4.55 ng/mL	10 (26.3)	60 (34.5)	
CEA_6w_	<3.41 ng/mL	27 (71.1)	94 (54.0)	0.070
	≥3.41 ng/mL	11 (28.9)	80 (46.0)	
CEA_12w_	<5.34 ng/mL	29 (76.3)	126 (72.4)	0.691
	≥5.34 ng/mL	9 (23.7)	48 (27.6)	
NSE_0w_	<22.30 ng/mL	31 (81.6)	154 (88.5)	0.282
	≥22.30 ng/mL	7 (18.4)	20 (11.5)	
NSE_6w_	<13.54 ng/mL	20 (52.6)	90 (51.7)	1.000
	≥13.54 ng/mL	18 (47.4)	84 (48.3)	
NSE_12w_	<14.55 ng/mL	20 (52.6)	116 (66.7)	0.135
	≥14.55 ng/mL	18 (47.4)	58 (33.3)	
Hb_0w_	<119.50 g/L	14 (36.8)	24 (13.8)	0.002
	≥119.50 g/L	24 (63.2)	150 (86.2)	
Hb_6w_	<117.50 g/L	10 (26.3)	31 (17.8)	0.258
	≥117.50 g/L	28 (73.7)	143 (82.2)	
Hb_12w_	<173.00 g/L	36 (94.7)	169 (97.1)	0.611
	≥173.00 g/L	2 (5.3)	5 (2.9)	
LDH_0w_	<187.50 U/L	22 (57.9)	82 (47.1)	0.283
	≥187.50 U/L	16 (42.1)	92 (52.9)	
LDH_6w_	<241.50 U/L	32 (84.2)	155 (89.0)	0.408
	≥241.50 U/L	6 (15.8)	19 (10.9)	
LDH_12w_	<191.50 U/L	18 (47.4)	90 (51.7)	0.721
	≥191.50 U/L	20 (52.6)	84 (48.3)	
WBC_0w_	<5.39×10^9^/L	5 (13.2)	32 (18.4)	0.637
	≥5.39×10^9^/L	33 (86.8)	142 (81.6)	
WBC_6w_	<9.34×10^9^/L	32 (84.2)	150 (86.2)	0.798
	≥9.34×10^9^/L	6 (15.8)	24 (13.8)	
WBC_12w_	<5.88×10^9^/L	11 (28.9)	83 (47.7)	0.047
	≥5.88×10^9^/L	27 (71.1)	91 (52.3)	
RBC_0w_	<5.81×10^12^/L	37 (97.4)	173 (99.4)	0.327
	≥5.81×10^12^/L	1 (2.6)	1 (0.6)	
RBC_6w_	<4.20×10^12^/L	17 (44.7)	75 (43.1)	0.859
	≥4.20×10^12^/L	21 (55.3)	99 (56.9)	
RBC_12w_	<3.81×10^12^/L	9 (23.7)	69 (39.7)	0.094
	≥3.81×10^12^/L	29 (76.3)	105 (60.3)	

**Figure 3:**
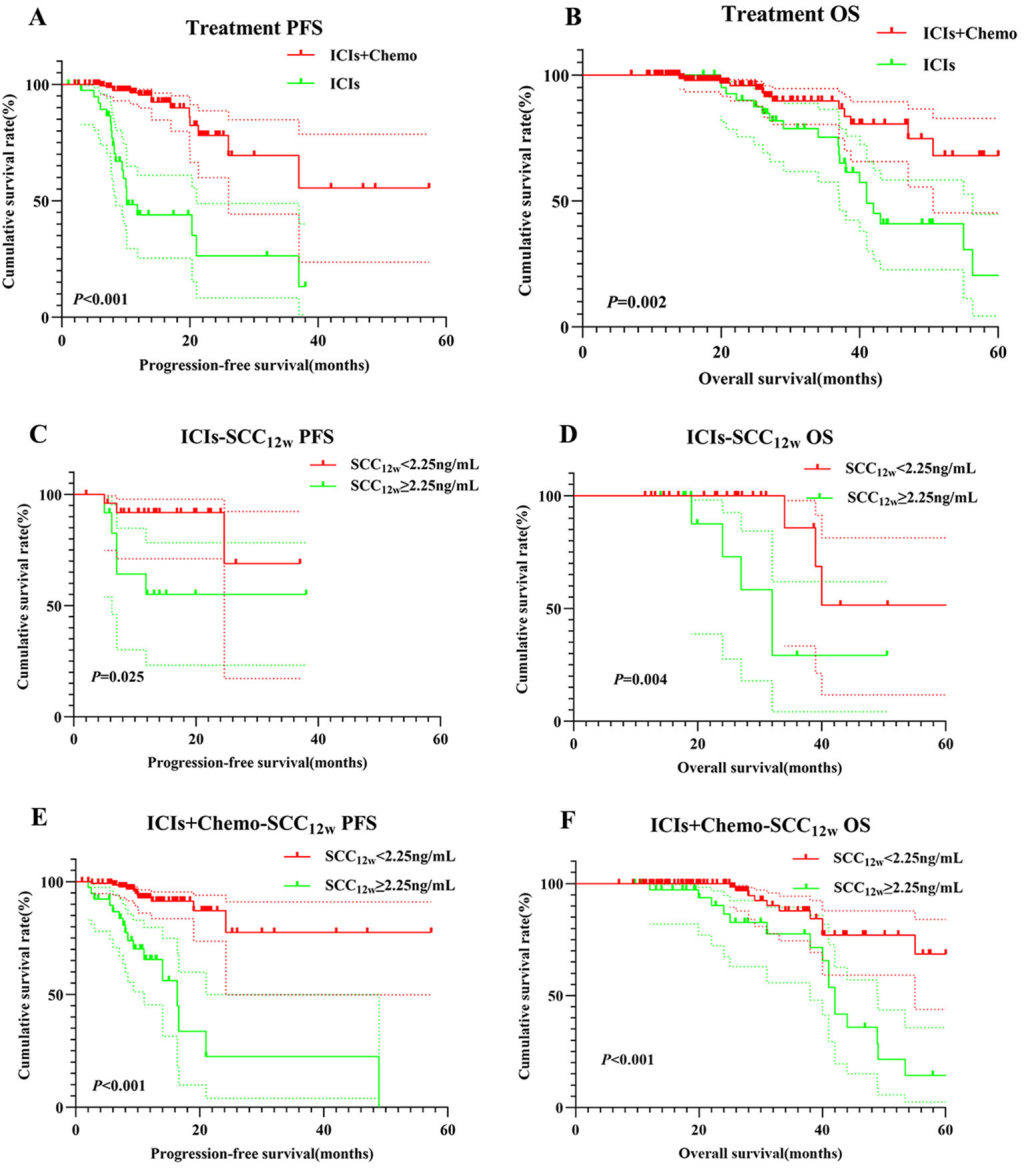
Survival curve for treatment and SCC_12w_.Treatment-related survival curve for **(A)** PFS and **(B)** OS; ICIs SCC_12w_-related survival curve for **(C)** PFS and **(D)** OS; ICIs+Chemo SCC_12w_-related survival curve for **(E)** PFS and **(F)** OS.

Patients in the ICIs group had significantly shorter PFS and OS compared to those in the ICIs+Chemo group (*p* < 0.001 *vs*. *p* = 0.002, **Figures 3A, B**). In the ICIs group, patients with SCC_12w_ < 2.25 ng/mL had a median PFS of 13.1 months and OS of 26 months, compared to 11.8 months and 19.9 months, respectively, in patients with SCC_12w_ ≥ 2.25 ng/mL (*p* = 0.025 *vs*. *p* = 0.004, **Figures 3C, D**). The 1- and 3-year PFS rates for patients with SCC_12w_ < 2.25 ng/mL were 86.8%(95% CI:0.836-0.912) and 68.9%(95% CI:0.809-0.921), respectively, while their OS rates were 83.7%(95% CI:0.633-0.901) and 68.6%(95% CI:0.403-0.857). In contrast, patients with SCC_12w_ ≥ 2.25 ng/mL had 1- and 3-year PFS rates of 82.8% (95% CI:0.801-0.890) and 62.8% (95% CI:0.420-0.937), and OS rates of 77.5% and 58.3%, respectively.

In the ICIs+Chemo group, patients with SCC_12w_ < 2.25 ng/mL had a median PFS of 12.4 months and OS of 26 months, whereas those with SCC_12w_ ≥ 2.25 ng/mL experienced significantly shorter PFS and OS (*p* < 0.001 *vs*. *p* < 0.001, **Figures 3E, F**). The 1- and 3-year PFS rates for patients with SCC_12w_ < 2.25 ng/mL were 86.8%(95% CI:0.836-0.912) and 68.9%(95% CI:0.809-0.921), while OS rates were 83.7%(95% CI:0.633-0.901) and 68.6%(0.403-0.857), respectively. By comparison, patients with SCC_12w_ ≥ 2.25 ng/mL had 1- and 3-year PFS rates of 82.1% (95% CI:0.5037-0.852) and 65.5% (95% CI:0.3714-0.706), and OS rates of 73.5% (95% CI:0.920-0.998) and 57.5%(95%CI:0.627-0.957), respectively.

### 3.8 Positive expression of programmed death ligand 1

**Figure 4:**
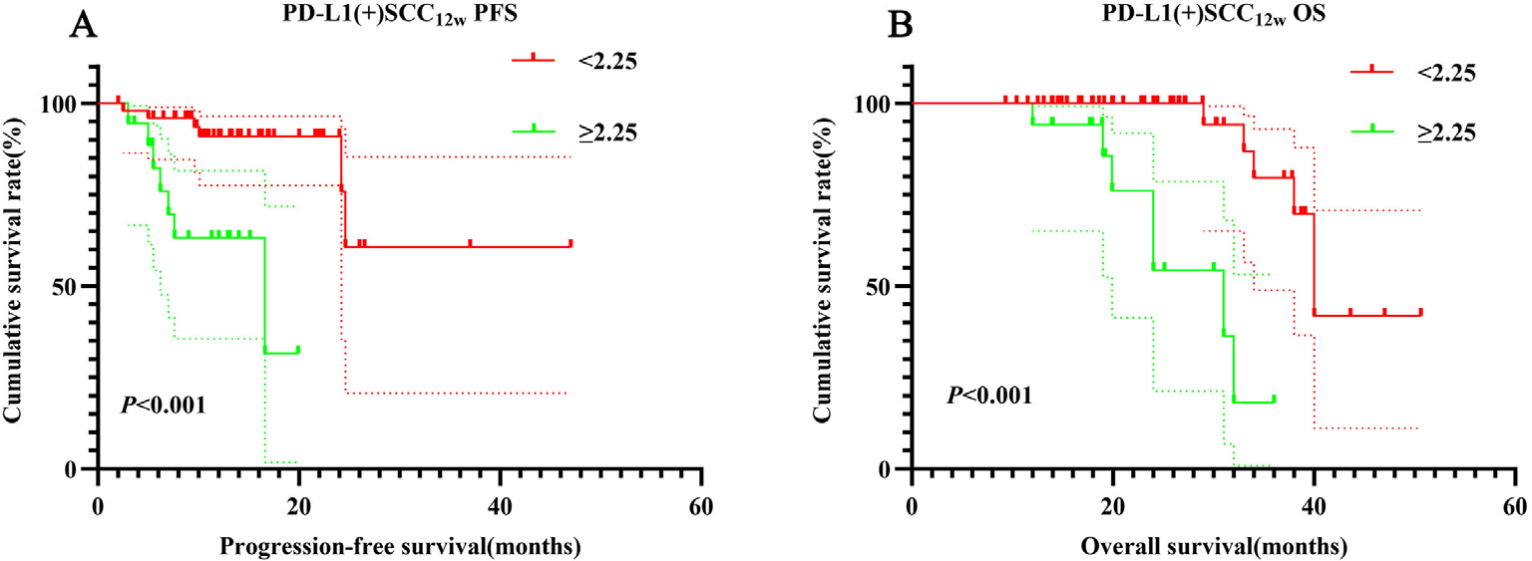
Survival curve for PD-L1(+) SCC_12w_. PD-L1(+) SCC_12w_-related survival curve for **(A)** PFS and **(B)** OS.

According to NCCN guidelines, assessing PD-L1 expression levels is recommended for patients receiving PD-1 inhibitors. PD-1/PD-L1 expression on tumor cells is analyzed using immunohistochemistry (IHC), with positivity defined as expression levels of ≥1%. In this retrospective study, PD-L1 expression was positive in 72 cases (33.9%), negative in 9 cases (4.2%), and unknown in 131 cases (61.8%). Among PD-L1-positive patients, those in the SCC_12w_ < 2.25 ng/mL group demonstrated significantly longer PFS and OS compared to the SCC_12w_ ≥ 2.25 ng/mL group (*p* < 0.001). The 1- and 3-year PFS and OS rates in the SCC_12w_ < 2.25 ng/mL group were 90.9% (95% CI:0.828-0.999)、60.6% (95% CI:0.342-1.000)、99.5%(95% CI:0.638-1.000) and 71.2%(95% CI:0.843-0.998), respectively, whereas these rates were markedly lower in the SCC_12w_ ≥ 2.25 ng/mL group at 58.7%(95% CI:0.392-0.878)、23.3%(95% CI:0.675-0.963)、94.1%(95% CI:0.836-1.000) and 29.1%(95% CI:0.097-0.873), respectively (**Figures 4A, B**).

### 3.9 Evaluation of the construction of column-line diagrams

**Figure 5:**
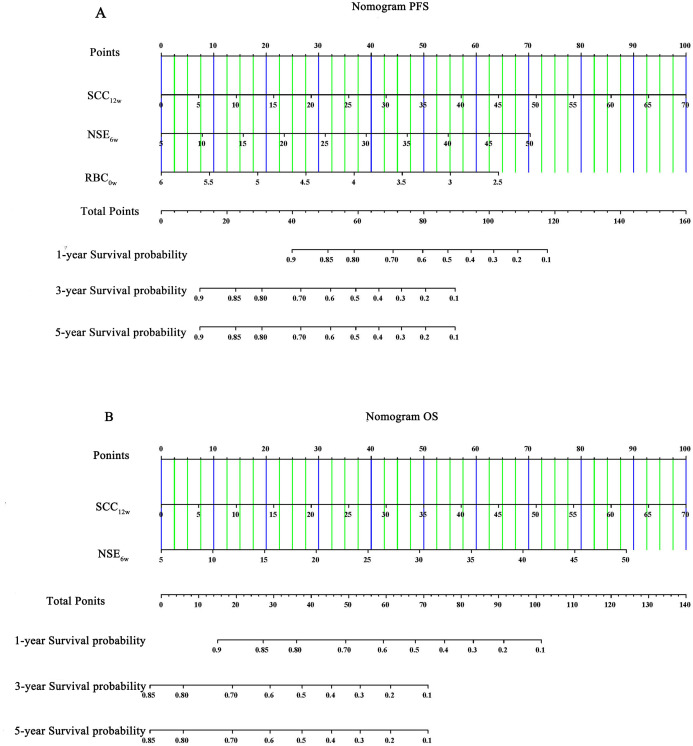
Nomogram of **(A)** PFS and **(B)** OS.

**Figure 6:**
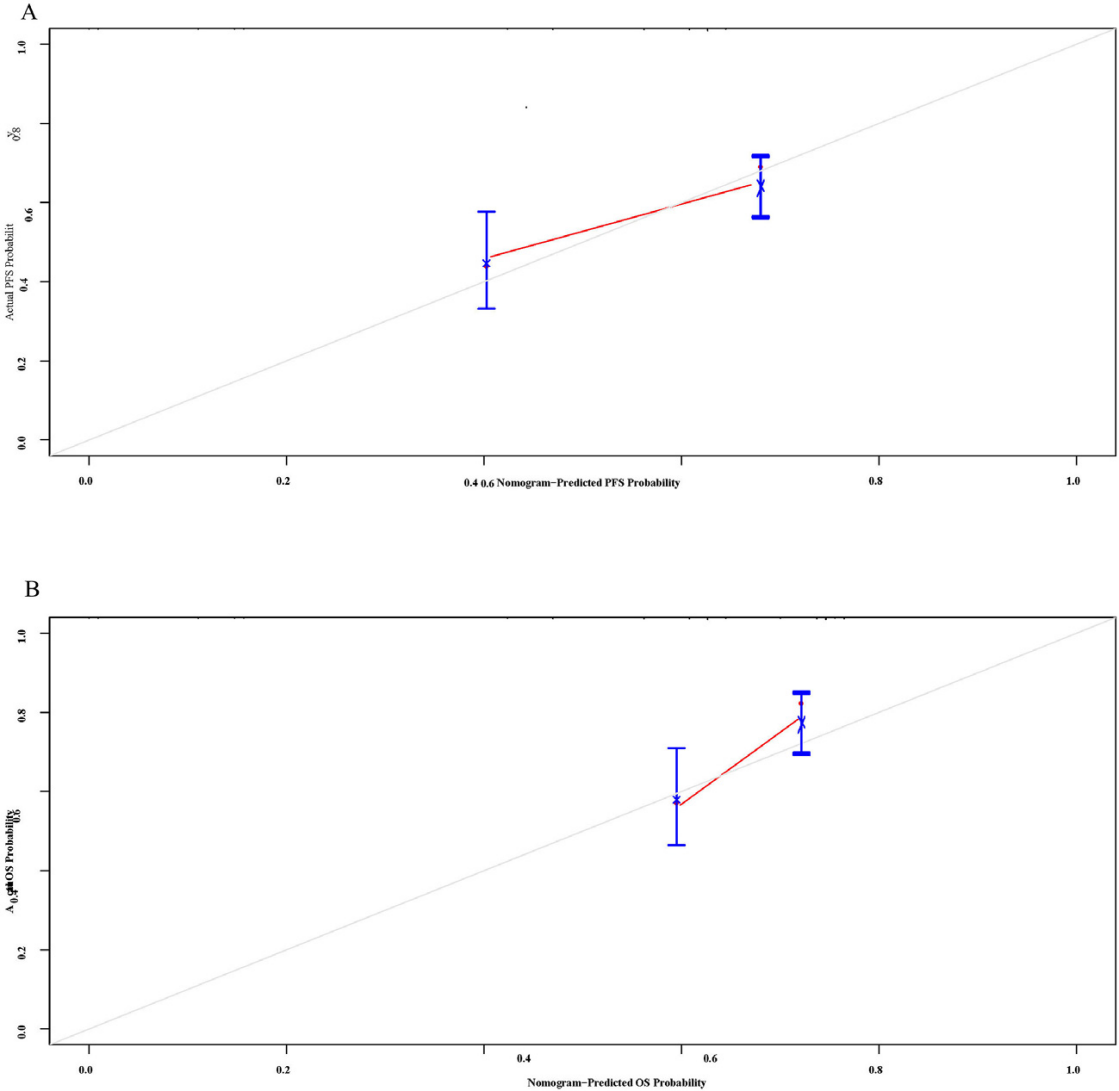
The calibration curves of the nomograms for **(A)** PFS and **(B)** OS.

Multivariate Cox regression analysis identified SCC_12w_, NSE_6w_, and RBC_0w_ as independent prognostic factors influencing the outcomes of ICIs. The Schenfeld residual test confirmed that these variables satisfied the proportional risk assumption for both PFS and OS analyses (*p* > 0.05, **Supplementary Figure 2**). To further evaluate their predictive accuracy, nomograms were developed to estimate the probability of PFS and OS at 1, 3, and 5 years (**Figures 5A, B**). The C-index values for the nomograms were 0.725 (95% CI: 0.478-0.928) and 0.755(95% CI: 0.642-0.868) for OS, indicating robust predictive performance. Additionally, Bootstrap correction demonstrated excellent consistency and calibration of the nomograms (**Figures 6A, B**).

## 4 Discussion

Chemotherapy, radiotherapy and targeted therapy have achieved some success in the treatment of lung cancer, but the efficacy is still unsatisfactory. In recent years, with the emergence of immunotherapy represented by immune checkpoint inhibitors has brought new hope to lung cancer patients (12). Anti-PD-1-based immunotherapy plays an important role in patients with advanced squamous lung cancer. Compared with conventional chemotherapy, PD-1 inhibitors have high efficacy in patients with advanced non-small cell lung cancer (12). PD-1 inhibitors alleviate the inhibitory effect of tumor cells on immune cells by blocking the binding of PD-L1 to PD-1, thus restoring the tumor-killing effect of the immune system (13). Although immunotherapy has revolutionized cancer treatment, less than 30% of patients benefit from ICIs (14). There is a lack of methods that can accurately predict the prognosis and predictors of immunotherapy. Identifying simple and affordable tools to predict the efficacy of immunotherapy in advanced cancer is one of the clinical needs that are currently being highly prioritized and researched.

Peripheral blood bioindicators are rapidly emerging in the field of immuno-oncology. They not only reflect tumor biology, but also provide evolving host immune responses to tumors. Han et al. found that TCR diversity of peripheral blood PD-1CD8+ T cells could be used as a non-invasive predictor of response to ICIs and survival outcomes in NSCLC patients (15). Increased PD-1CD8+ TCR clonality 4-6 weeks after ICIs treatment was associated with higher DCR, longer PFS and OS (15). In addition, peripheral blood inflammatory markers, such as NLR and LDH (16), can predict the prognosis of immunotherapy in patients with advanced NSCLC (17). Higher-than-normal levels of C-reactive protein have been associated with poorer PFS and OS in patients with PD-1 inhibitor-treated cancers (18). In a retrospective study of 1714 cases of 16 different cancer types treated with ICIs, the combination of low NLR/high TMB group was found to provide significant benefit from ICIs therapy (19). Dynamic changes in peripheral blood inflammatory biomarkers also reflect treatment response and prognosis in NSCLC patients receiving neoadjuvant immunotherapy (20). Not only that, tumor markers such as α-fetoprotein (21) and CEA (22) are helpful in clinical assessment of PD-1 inhibitor efficacy and predictive modeling. In a multicenter retrospective analysis, CRP and alpha-fetoprotein were found to score immunotherapy in patients with hepatocellular carcinoma treated with atezolizumab in combination with bevacizumab, and patients with AFP ≥ 100 ng/mL and CRP ≥1 mg/dL were found to have a poorer prognosis (21). In several retrospective analyses, it was found that a reduction in CEA or CYFRA21-1 levels may be a reliable biologic predictor of the efficacy of immunotherapy in patients with NSCLC (22).

Because of the close relationship between tumor markers and squamous cell carcinoma of the lung, we first linked several common tumor markers to the prognosis of immunotherapy for squamous cell carcinoma of the lung. We found that RBC and WBC were predictive of immunotherapy response, with RBC below 3.81 × 10^12^/L at week 12 associated with lower ORR; WBC below 9.34 × 10^9^/L at week 6 post-treatment was associated with higher DCR. In an extensive analysis of the prognostic value of various parameters, we found that SCC_12w_ had the highest AUC among the blood markers. Not only that, SCC_12w_ was also an independent predictor of survival prognosis in advanced squamous lung carcinoma treated with PD-1 inhibitors. In addition, the analysis of the survival outcome showed that SCC_12w_ higher than 2.25 ng/mL was associated with a poorer prognosis, which was in line with Zhang et al. concluded that lower combined levels of SCC-Ag, CEA, CA125, and CYFRA21-1 were consistent with the result that immunotherapy for advanced squamous lung cancer had better survival outcomes (9). In our analysis, NSE was also an independent predictor of prognostic indicators for advanced squamous lung cancer treated with PD-1 inhibitors. Huang et al. found that serum CEA, NSE and CYFRA21-1 can predict PFS and OS in patients with advanced LUAD and LUSC. The higher the serum levels of NSE, CYFRA21-1 and CA125, the worse the PFS and OS in patients with LUAD and LUSC (23). According to NCCN guidelines, patients with positive PD-L1 expression usually show a better prognosis in terms of immunotherapy. However, in this study, there were only 9 patients with negative PD-L1 expression, which is a small sample size. Preliminary analysis showed possible bias error. Therefore, we excluded patients with PD-L1-negative expression and included only patients with PD-L1-positive expression for prognostic analysis. We evaluated the predictive value of hematological indices in patients with positive PD-L1 expression for the prognosis of survival after treatment. The results showed that SCC_12w_ above 2.25 ng/mL and NSE_6w_ below 13.54 ng/mL had a poor prognosis among patients with positive PD-1 expression after treatment.

Our study still has limitations. First, this study was a retrospective analysis with a small sample size, and more lung cancer patients treated with ICIs and multicenter prospective cohort studies should be recruited. Second, the lack of effective information on PD-L1 in patients, our findings may differ from the real world may. In fact, PD-L1 showed some predictive effects in non-small cell lung cancer treated with ICIs. Third, there were different time points of patients’ treatment with ICIs, and there was heterogeneity in this study as only 0, 6, and 12 weeks of treatment were included.

## 5 Conclusion

In conclusion, our findings indicate that post-treatment SCC and NSE reduction serves as an independent prognostic factor for PD-1 inhibitor therapy in patients with advanced squamous lung cancer. Furthermore, our results demonstrate a significant association between SCC and NSE reduction and improved prognosis in patients treated with PD-1 inhibitors.

## Data Availability

The raw data supporting the conclusions of this article will be made available by the authors, without undue reservation.
