# Supplementary figures and images for: The establishment of PD-1 inhibitor treatment prognosis model based on dynamic changes of peripheral blood indexes in patients with advanced lung squamous cell carcinoma
Source: Front Oncol. 2024 Dec 17;14:1454709. doi: 10.3389/fonc.2024.1454709 (PMC11685080; doi:10.3389/fonc.2024.1454709)

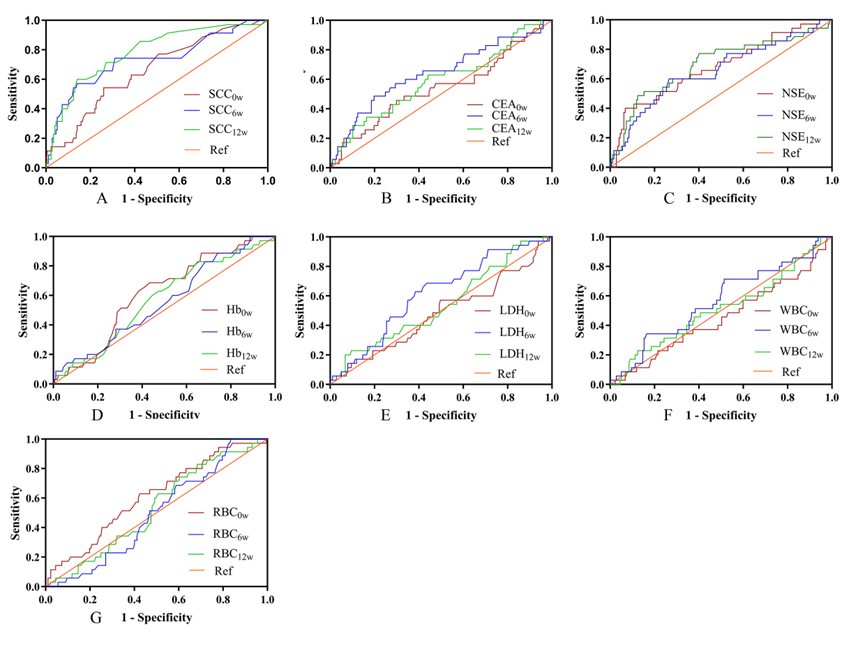


Supplementary Figure 1 The ROC curve of (A)SCC;(B) CEA;(C) NSE;(D) Hb;(E）LDH;(F) WBC;(G) RBC

Supplement: Supplementary file 1 [file DataSheet1.docx]

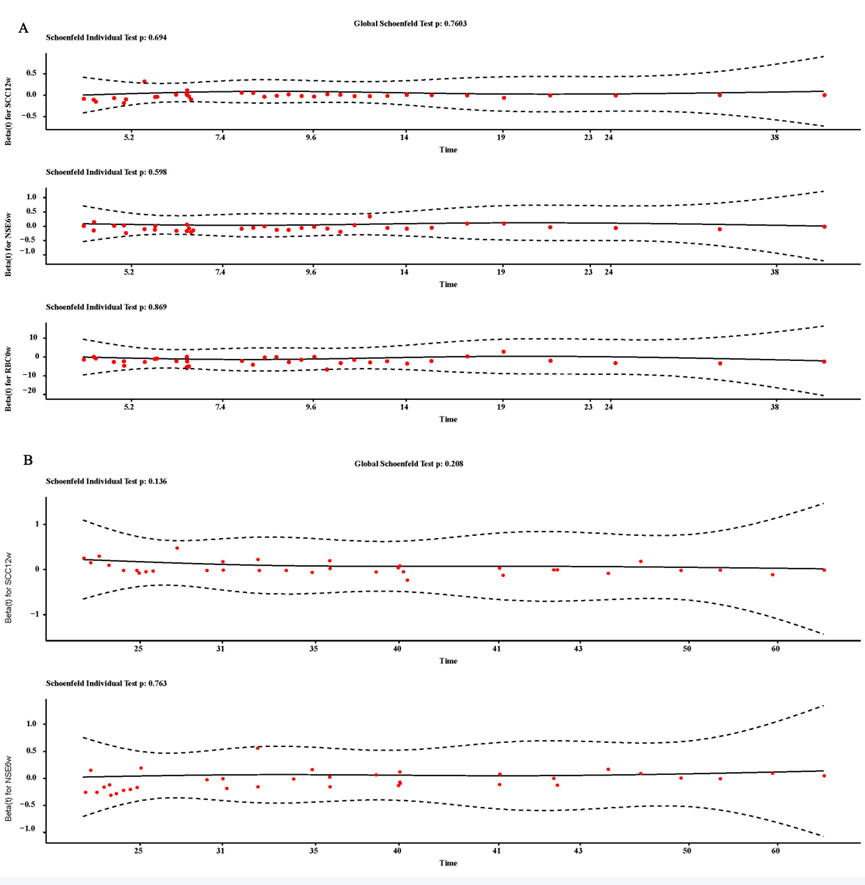


Supplementary figure2: The Schenfeld individual test of the nomograms for (A) PFS and (B) OS.

Supplement: Supplementary file 2 [file DataSheet2.docx]
